# The organizer of chromatin topology RIF1 ensures cellular resilience to DNA replication stress

**DOI:** 10.26508/lsa.202101186

**Published:** 2023-02-06

**Authors:** Rana Lebdy, Julie Patouillard, Marion Larroque, Serge Urbach, Raghida Abou Merhi, Christian Larroque, Cyril Ribeyre

**Affiliations:** 1 Institut de Génétique Humaine , CNRS UMR9002, Université de Montpellier, Montpellier, France; 2 Institut du Cancer de Montpellier, Montpellier, France; 3 Institut de Génomique Fonctionnelle, CNRS UMR5203, INSERM U1191, Université de Montpellier, Montpellier, France; 4 Doctoral School of Sciences and Technology-DSST, Rafic Hariri Campus, Lebanese University, Hadath, Lebanon; 5 Institut de Recherche en Cancérologie de Montpellier, INSERM U1194, Université de Montpellier, Montpellier, France

## Abstract

Eukaryotic genomes are duplicated from thousands of replication origins that fire sequentially forming a defined spatiotemporal pattern of replication clusters. The temporal order of DNA replication is determined by chromatin architecture and, more specifically, by chromatin contacts that are stabilized by RIF1. Here, we show that RIF1 localizes near newly synthesized DNA. In cells exposed to the DNA replication inhibitor aphidicolin, suppression of RIF1 markedly decreased the efficacy of isolation of proteins on nascent DNA, suggesting that the isolation of proteins on nascent DNA procedure is biased by chromatin topology. RIF1 was required to limit the accumulation of DNA lesions induced by aphidicolin treatment and promoted the recruitment of cohesins in the vicinity of nascent DNA. Collectively, the data suggest that the stabilization of chromatin topology by RIF1 limits replication-associated genomic instability.

## Abstract (precis)

The conserved protein RIF1 stabilizes replication factories and regulates cohesin recruitment in response to replication stress to limit DNA lesions.

## Introduction

The duplication of a complete genome is a formidable task that must be perfectly controlled to avoid the transmission of mutations or chromosomal rearrangements to daughter cells. 2 m of DNA is packed and replicated in a human cell of about 10 μm diameter. Hence, the spatiotemporal program of DNA replication is largely defined by the global organization of the nucleus. DNA replication is initiated from defined regions of the genome called origins of replication. More than 30,000 replication origins are required for the duplication of the human genome (Mechali, 2010). When replication forks stall, the firing of backup origins (also known as dormant origins) ensures the completion of DNA replication (Blow et al, 2011). The timing of replication is influenced by the 3D organization of chromatin architecture (Courbet et al, 2008; Foti et al, 2016; Klein et al, 2021). Cohesin influence origins firing locally (Guillou et al, 2010), yet without determining replication timing globally (Oldach & Nieduszynski, 2019), most likely via the formation of loops by extrusion (Davidson et al, 2019; Kim et al, 2019). RIF1, a conserved protein involved in telomere capping, DNA double-strand break (DSB) repair, and chromatin organization, controls the timing of DNA replication (Cornacchia et al, 2012; Hayano et al, 2012; Yamazaki et al, 2012; Foti et al, 2016; Mattarocci et al, 2016; Klein et al, 2021; Richards et al, 2022). RIF1 determines replication timing via the stabilization of chromatin architecture (Yamazaki et al, 2013; Kanoh et al, 2015; Foti et al, 2016; Klein et al, 2021) and may regulate origin licensing owing to its interaction with PP1 phosphatase that would counteract DDK kinases (Dave et al, 2014; Hiraga et al, 2014; Mattarocci et al, 2014).

Throughout the S phase, different nuclear patterns of replication foci reflect the orderly and sequential replication of chromatin domains (Dimitrova & Berezney, 2002; Chagin et al, 2016). Replication forks encounter a variety of impediments from both endogenous and exogenous sources (Lambert & Carr, 2013; Zeman & Cimprich, 2014). The slowing or stalling of replication forks by these impediments induces the activation of the checkpoint kinase ATR, which ensures that DNA synthesis within actively replicating chromosomal domains is completed before the duplication of a new chromosomal domain has started. ATR signaling delays the activation of late replication domains while promoting the firing of dormant origins within active replication domains (Blow et al, 2011). This suggests that the nuclear architecture contributes to cellular resilience to DNA replication stress. In support of this, Lamin A/C is required for the maintenance of chromosome integrity when the progression of replication forks is impeded by DNA lesions or upon nucleotide depletion (Singh et al, 2013). Furthermore, the association of Lamin A/C with the DNA polymerase clamp PCNA is critical for replication fork stability (Cobb et al, 2016). Hutchinson–Gilford progeria syndrome is caused by a mutation of the *LMNA* gene that leads to an aberrant Lamin A protein named progerin. The association of progerin with PCNA alters the nuclear distribution of PCNA, and induces ATR activation and the formation of γH2A.X (Wheaton et al, 2017). In budding yeast, cohesin accumulates in the vicinity of replication forks upon treatment with hydroxyurea and is required for replication fork restart (Tittel-Elmer et al, 2012). These examples illustrate the links between replication stress and nuclear structures, which remain incompletely understood.

The isolation of proteins on nascent DNA coupled with mass spectrometry (iPOND-MS) allows the identification of proteins localized in the vicinity of active replication forks (Sirbu et al, 2011, 2013; Lopez-Contreras et al, 2013; Lossaint et al, 2013; Aranda et al, 2014; Dungrawala et al, 2015). iPOND experiments performed under various experimental conditions have revealed components of the replication machinery (e.g., PCNA and DNA polymerases), proteins that accumulate near forks under stressful conditions (e.g., ATR and FANCD2), proteins that are required for the restoration of chromatin structures after passage of the replication fork (e.g., histones), and proteins that are playing structural roles such as Lamin A (Sirbu et al, 2011, 2013; Lopez-Contreras et al, 2013; Lossaint et al, 2013; Alabert et al, 2014; Dungrawala et al, 2015; Ribeyre et al, 2016; Wheaton et al, 2017).

Here, we provide evidence that during the S phase, RIF1 is proximal to newly synthesized DNA. In cells exposed to the DNA polymerase inhibitor aphidicolin, RIF1 promotes the recruitment of the cohesin subunits SMC1 and SMC3 near replication forks and stabilizes replicating nucleoprotein clusters isolated by iPOND. We propose that the stabilization of chromatin architecture by RIF1 and cohesin limits the formation of DNA lesions caused by DNA replication impediments.

## Results

### iPOND coupled with mass spectrometry identifies proteins involved in nuclear organization

**Figure 1.**
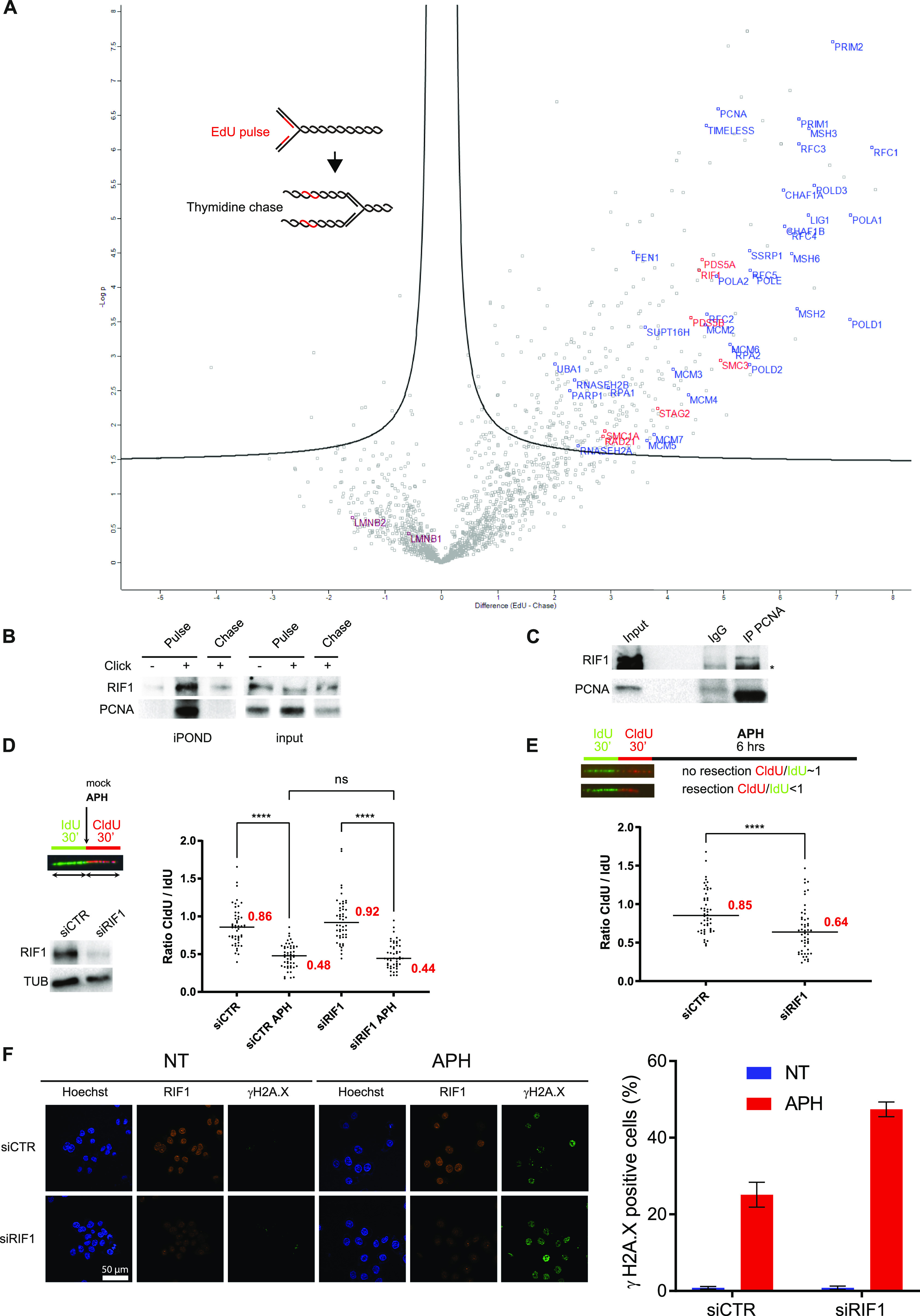
RIF1 is associated with nascent DNA and is required to limit DNA lesions in response to prolonged aphidicolin treatment. **(A)** iPOND coupled with mass spectrometry. HeLa S3 cells were pulse-labeled with EdU or pulse-labeled with EdU followed by a 120-min thymidine chase, then subjected to iPOND, and analyzed by mass spectrometry. Label-free quantification was performed using MaxQuant (Cox & Mann, 2008) and statistical analysis using Perseus (Tyanova et al, 2016). Pulse experiments have been performed six times and chase experiments four times. Examples of replisome-specific proteins are indicated on the right side of the figure above the line. Full protein list is available in Table S1. **(B)** Indicated proteins were isolated by iPOND and detected by Western blotting. HeLa S3 cells were pulse-labeled with EdU for 15 min and chased with thymidine for 120 min. In no-click samples, biotin–TEG azide was replaced by DMSO. **(C)** Western blot analysis of indicated proteins after immunoprecipitation with an antibody directed against PCNA or against mouse IgG. **(D)** DNA fiber labeling and Western blot analysis of RIF1 depletion. HeLa S3 cells were labeled for 30 min with IdU and then for 30 min with CldU in the absence or presence of 0.05 μM aphidicolin (APH) in the cell culture medium. Graphic representation of the ratios of CldU versus IdU tract length. For statistical analysis, a Mann–Whitney test was used, ns, non-significant; ****P < 0.0001. The horizontal bar represents the median with the value indicated in red. 50 replication tracts were measured for each experimental condition. **(E)** Analysis of DNA resection using DNA fiber labeling. HeLa S3 cells were labeled for 30 min with IdU and then for 30 min with CldU. 1 μM aphidicolin (APH) was added in the cell culture medium for 6 h. Graphic representation of the ratios of CldU versus IdU tract length. For statistical analysis, a Mann–Whitney test was used, ****P < 0.0001. The horizontal bar represents the median with the value indicated in red. 50 replication tracts were measured for each experimental condition. **(F)** Immunofluorescence analysis of γH2A.X and RIF1 in HeLa S3 cells with siRNA against control or RIF1 in the presence or absence of aphidicolin (APH) for 24 h. Graphic representation of the percentage of γH2A.X–positive cells based on three independent experiments.

To identify new proteins in the vicinity of replication forks, we performed iPOND-MS using a highly sensitive last-generation mass spectrometer (SCIEX TripleTOF 5600+) and quantified the results using MaxQuant (Cox & Mann, 2008). We analyzed the data using Perseus (Tyanova et al, 2016) and took advantage of a volcano plot representation to visualize the proteins significantly enriched upon the EdU pulse compared with a 2-h thymidine chase (Fig 1A). As expected, most of the known proteins of the replisome (e.g., PCNA, RFC subunits, MCM1-6, FEN1, or DNA polymerases) were clearly enriched. We also identified many proteins that were not previously described as replisome components that will be analyzed elsewhere. Interestingly, the iPOND-MS data revealed an enrichment of several cohesin subunits (SMC3, SMC1A, STAG2, RAD21, PDS5A, and PDS5B) near forks (Fig 1A). Because cohesins are thought to play an architectural role at replication foci (Guillou et al, 2010), it is likely that they are not associated directly with individual replication forks but rather with chromatin domains undergoing replication. In contrast, Lamin B1 and Lamin B2 were not enriched after the EdU pulse (Fig 1A), indicating that not all the structural components of the nucleus are localized in the proximity of active replisomes. Interestingly, we identified RIF1 as a protein associated with nascent DNA (Fig 1A), consistent with previous studies (Alabert et al, 2014; Munden et al, 2018). We confirmed these data using an antibody directed against RIF1 (Fig 1B). This indicates that RIF1 localizes in the vicinity of active replication forks. Consistent with this, we detected RIF1 in immunoprecipitates of the endogenous DNA polymerase clamp PCNA (Fig 1C).

Table S1 Perseus analysis of proteins identified in iPOND-MS analysis upon pulse-chase experiments.

Table S2 Label free quantification of known replisome components identified in iPOND-MS upon RIF1 depletion and aphidicolin treatment.

### RIF1 protects the integrity of replication forks upon prolonged replication stress

**Figure S1.**
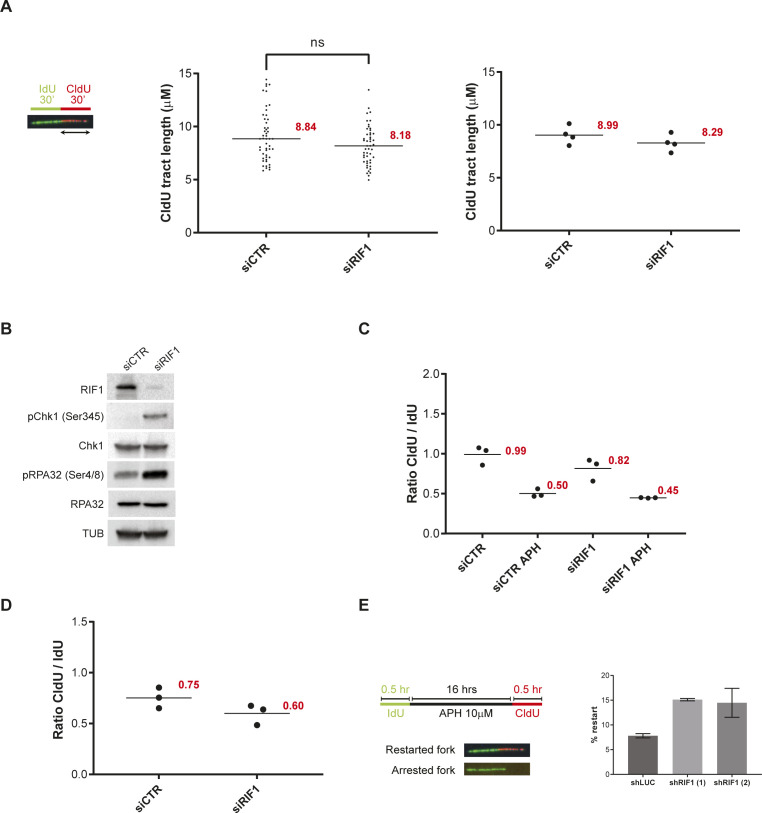
Impact of RIF1 depletion on replication stress. **(A)** DNA fiber labeling. HeLa S3 cells were labeled for 30 min with IdU and then for 30 min with CldU. Graphic representation of the ratios of CldU tract length. The horizontal bar represents the median with the value indicated in red. For statistical analysis, a Mann–Whitney test was used, ns, non-significant. At least 50 replication tracts were measured for each experimental condition. The second graphic representation is showing the average of four independent experiments. **(B)** Western blot analysis of the indicated proteins upon transfection with siRNA directed against RIF1 or a control target. **(C)** This graphic representation is showing the average of the three independent experiments from Fig 1D. **(D)** This graphic representation is showing the average of the three independent experiments from Fig 1E. **(E)** Analysis of replication restart upon APH treatment using DNA fiber labeling. HeLa S3 cells were labeled for 30 min with IdU, then treated 16 h with 10 μM APH and then for 30 min with CldU. Graphic representation of the percentage of restart based on three independent experiments.

Although RIF1 is located near active replisomes, suppression of RIF1 did not significantly alter the progression of replication forks (Fig S1A), consistent with previous studies (Cornacchia et al, 2012; Ray Chaudhuri et al, 2016). A higher frequency of stalled forks, however, was observed in *rif1*^−/−^ DT40 cells (Xu et al, 2010), suggesting that RIF1 could be important for fork progression in some contexts. Consistent with this, several studies have detected the activation of the checkpoint effector kinase Chk1 in RIF1-depleted cells (Chapman et al, 2013; Foti et al, 2016). We confirmed that Chk1 was active by phosphorylation on serine 345 upon suppression of RIF1 by means of siRNAs (Fig S1B). We observed also that RPA32 was phosphorylated on Ser4/8, suggesting that RIF1-depleted cells accumulate DNA lesions (Fig S1B). Interestingly, RIF1 recruitment at replication forks is slightly increased upon hydroxyurea (HU) treatment to limit DNA2-mediated DNA resection and DNA lesions (Ray Chaudhuri et al, 2016; Garzón et al, 2019; Mukherjee et al, 2019). Consistent with this, DNA lesions, genetic instability, and HU sensitivity are increased upon RIF1 impairment (Buonomo et al, 2009; Xu et al, 2010; Mukherjee et al, 2019). This raises the possibility that the stabilization of chromatin topology by RIF1 limits replication-associated DNA lesions under stressful conditions. To test this, we analyzed whether RIF1 loss had any impact on replication fork dynamics in the presence of aphidicolin (APH). We labeled cells for 30 min with IdU and then for 30 min with CldU in the presence of a low dose (0.05 μM) of APH. As expected, the ratio of the lengths of CldU versus IdU tracts was close to 1 in control conditions and reduced by half in the presence of APH (Figs 1D and S1C). The status of RIF1 did not change the ratios of CldU to IdU tracts (Figs 1D and S1C), indicating that RIF1 depletion does not play any major role in early responses to APH. As RIF1 is protecting HU-stalled forks from nuclease degradation (Garzón et al, 2019; Mukherjee et al, 2019), we tested whether this was also the case when replication forks were blocked with APH. To do so, we treated cells 6 h with a high dose (1 μM) of APH after 30-min sequential labeling of IdU and CldU and measured the ratio between the lengths of CldU and IdU tracts. The ratio was close to 1 in cells treated with a control siRNA, and below 1 in RIF1-depleted cells, confirming that RIF1 is indeed protecting APH-stalled forks (Figs 1E and S1D). Consistent with this, prolonged treatment (24 h) with APH increased the percentage of γ-H2A.X–positive cells to almost twofold (Fig 1F) and decreased by twofold the ability of replication forks to restart (Fig S1E). Altogether, these data indicate that RIF1 limits the formation of DNA lesions under stressful conditions.

### RIF1-dependent loss of replication organization induces DNA lesions

**Figure 2.**
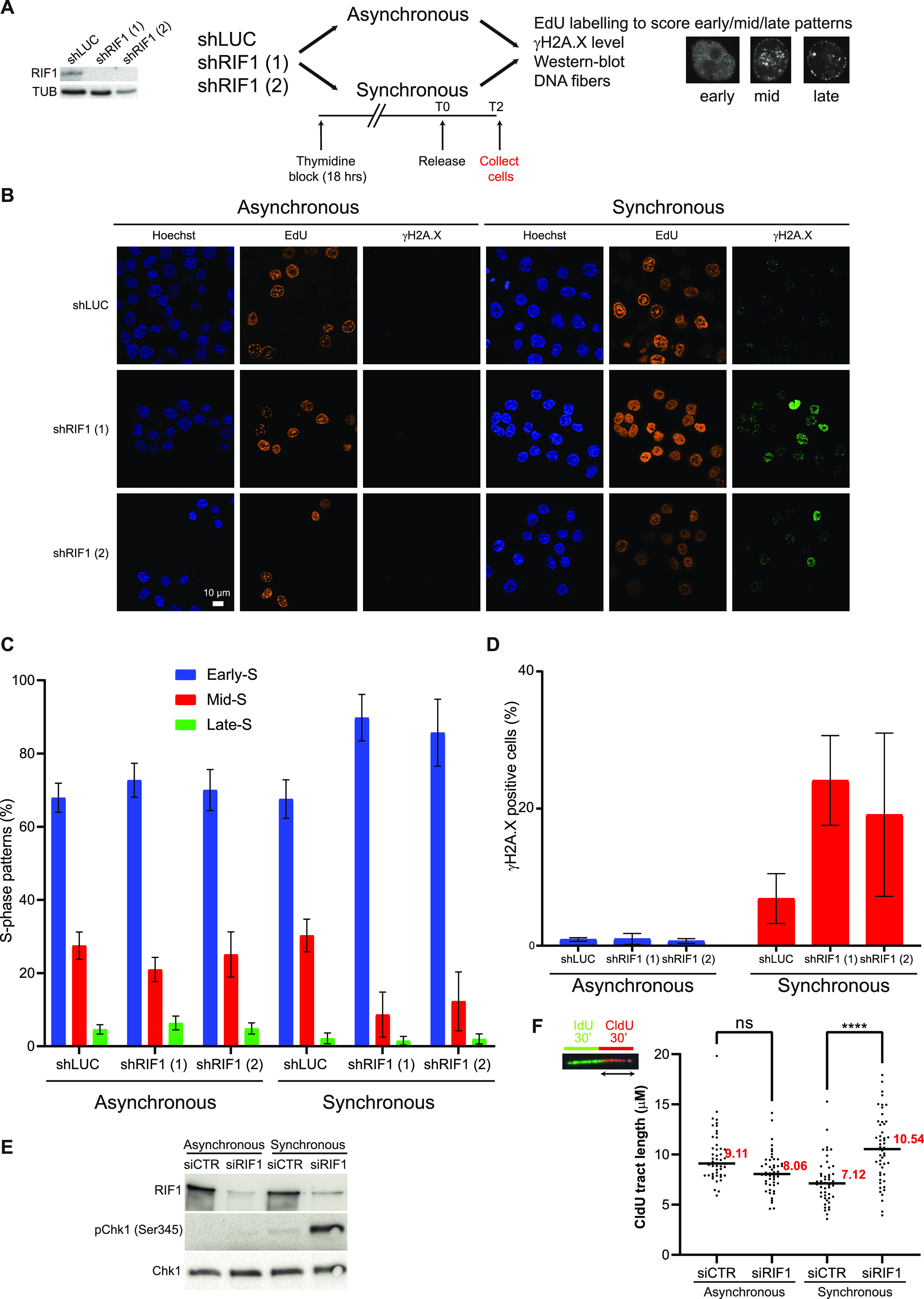
RIF1 depletion alters S-phase organization and yields DNA lesions. **(A)** Experimental setup to study the impact of synchronization procedure in HeLa S3 cells depleted or not for RIF1. The efficacy of RIF1 depletion using two different shRNAs is shown. For synchronization, cells were grown 18 h in the presence of 2 mM thymidine, then released into the S phase for 2 h. Cells were then subjected to immunofluorescence, Western blot, or DNA fiber analyses. **(B)** Immunofluorescence analysis of γH2A.X and EdU in asynchronous and synchronous HeLa S3 cells expressing shRNAs against luciferase or RIF1. **(C)** Graphic representation of the frequency of replication patterns (Late-S, Mid-S, and Early-S) based on at least three independent experiments for each condition. **(D)** Quantification of γH2A.X intensity within nucleus stained with Hoechst using CellProfiler based on at least three independent experiments for each condition. **(E)** Western blot analysis of Chk1 phosphorylation on serine 345 upon RIF1 depletion. **(F)** DNA fiber assay. HeLa S3 cells were labeled for 30 min with IdU and then for 30 min with CldU. Graphic representation of CldU tract lengths. For statistical analysis, a Mann–Whitney test was used, ns, non-significant; ****P < 0.0001. The horizontal bar represents the median with the value indicated in red. At least 50 replication tracts were measured for each experimental condition.

**Figure S2.**
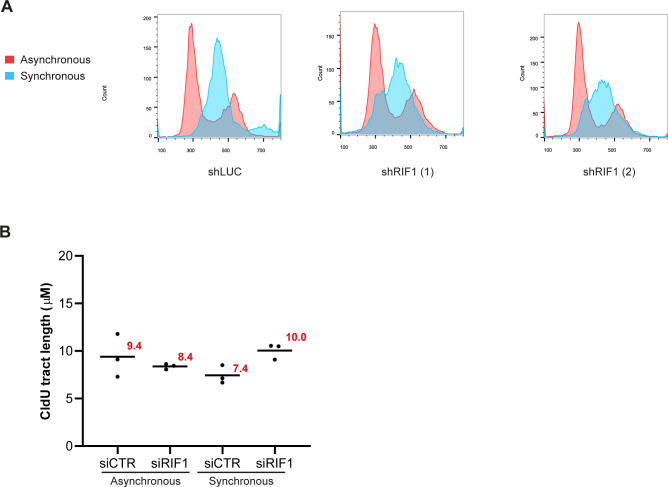
Impact of RIF1 depletion on replication forks dynamic upon synchronization in G1 and release into S-phase. **(A)** Flow cytometry analysis of cells depleted or not for RIF1 in asynchronous or synchronous conditions (18-h thymidine block followed by 2-h release). DNA was stained using propidium iodide. **(B)** This graphic representation is showing the average of the three independent experiments from Fig 2F.

Despite its role in protection of stalled replication forks (see above), RIF1 recruitment at forks does not increase drastically in response to HU (Mukherjee et al, 2019) compared with proteins such as ATR, 9-1-1, TopBP1, or FANCD2/FANCI (Lossaint et al, 2013; Dungrawala et al, 2015). Therefore, we hypothesize that the impact of RIF1 on nascent DNA protection may not reflect a direct role at stalled replication forks. This is supported by several articles, showing that RIF1 is crucial for the organization of higher order chromatin domains and for the establishment of the replication timing program (Yamazaki et al, 2012; Foti et al, 2016; Moriyama et al, 2018; Klein et al, 2021). Remarkably, the mid-S pattern is selectively lost upon RIF1 impairment (Yamazaki et al, 2012); this effect was attributed to the impact of RIF1 in replication timing. However, we noticed that these experiments have been performed in cells synchronized with a thymidine block and released into the S phase. It is well established that synchronization with the thymidine block perturbs the pool of nucleotides and induces DNA damage (Kurose et al, 2006). Thus, we hypothesized that the absence of the mid-S pattern in RIF1-depleted cells synchronized using a thymidine block could reflect a defect in the maintenance of chromatin topology during DNA replication stress. To test this, we compared the frequency of each pattern in asynchronous conditions and in cells synchronized with the thymidine block and released into the S phase upon RIF1 depletion (Fig 2A). In the synchronous condition, we were able to reproduce the results of Yamazaki et al and observed the disappearance of the mid-S pattern upon RIF1 depletion (Fig 2B and C). Surprisingly, in asynchronous conditions, we found that RIF1 depletion did not alter the occurrence of the mid-S pattern (Fig 2B and C). Importantly, and as already observed (Yamazaki et al, 2012), cell-cycle distribution was not significantly affected in the absence of RIF1 in synchronous or asynchronous conditions (Fig S2A). This result suggests that the disappearance of the mid-S pattern in RIF1-depleted cells is a consequence of the synchronization procedure and cannot be solely explained by the difference in replication timing because it should be also observed in asynchronous cells. To test whether synchronization procedure increases the level of replication stress, we analyzed the level of the marker of DNA damage γ-H2A.X. In an asynchronous population of cells, the depletion of RIF1 had no impact on the percentage of γ-H2A.X–positive cells (Fig 2B and D). As expected, the percentage of γ-H2A.X–positive cells increased 2 h after release from the thymidine block. Strikingly, inactivation of RIF1 tripled the percentage of γ-H2A.X–positive cells in the same conditions (6.9% in control versus 24.1% in shRIF1 (1) and 19.1% in shRIF1 (2)). We conclude that the disappearance of the mid-S pattern upon RIF1 depletion correlates with the formation of DNA lesions. However, we cannot rule out that synchronization of control cells leads to an enrichment of cells in mid-S compared with cells depleted for RIF1 as suggested by minor differences in cell-cycle distribution (Fig S2A). The thymidine block procedure is affecting the pool of dNTPs and therefore should have a direct impact on the progression of replication forks that might be exacerbated in the absence of RIF1. To test this, we monitored the phosphorylation of Chk1 on serine 345. In the control condition, we observed a mild phosphorylation of Chk1 on serine 345, in line with the higher level of γ-H2A.X (Fig 2E). Interestingly, we observed a strong level of Chk1 phosphorylation in RIF1-depleted cells 2 h after release from the thymidine block (Fig 2E). In asynchronous conditions, suppression of RIF1 did not significantly alter the progression of replication forks (Fig S1A). Two hours after release from a thymidine block, however, replication tracts were longer in the absence of RIF1 (Figs 2F and S2B). Unrestrained DNA synthesis would yield single-stranded DNA gaps detected and signaled by ATR, consistent with a higher level of Chk1 and H2A.X phosphorylation. We propose that the occurrence of DNA lesions during prolonged replication stress observed in RIF1-depleted cells is a consequence of alterations in the organization of replicated chromatin domains.

### RIF1 impairment reduces iPOND efficiency in the presence of replication stress

**Figure 3.**
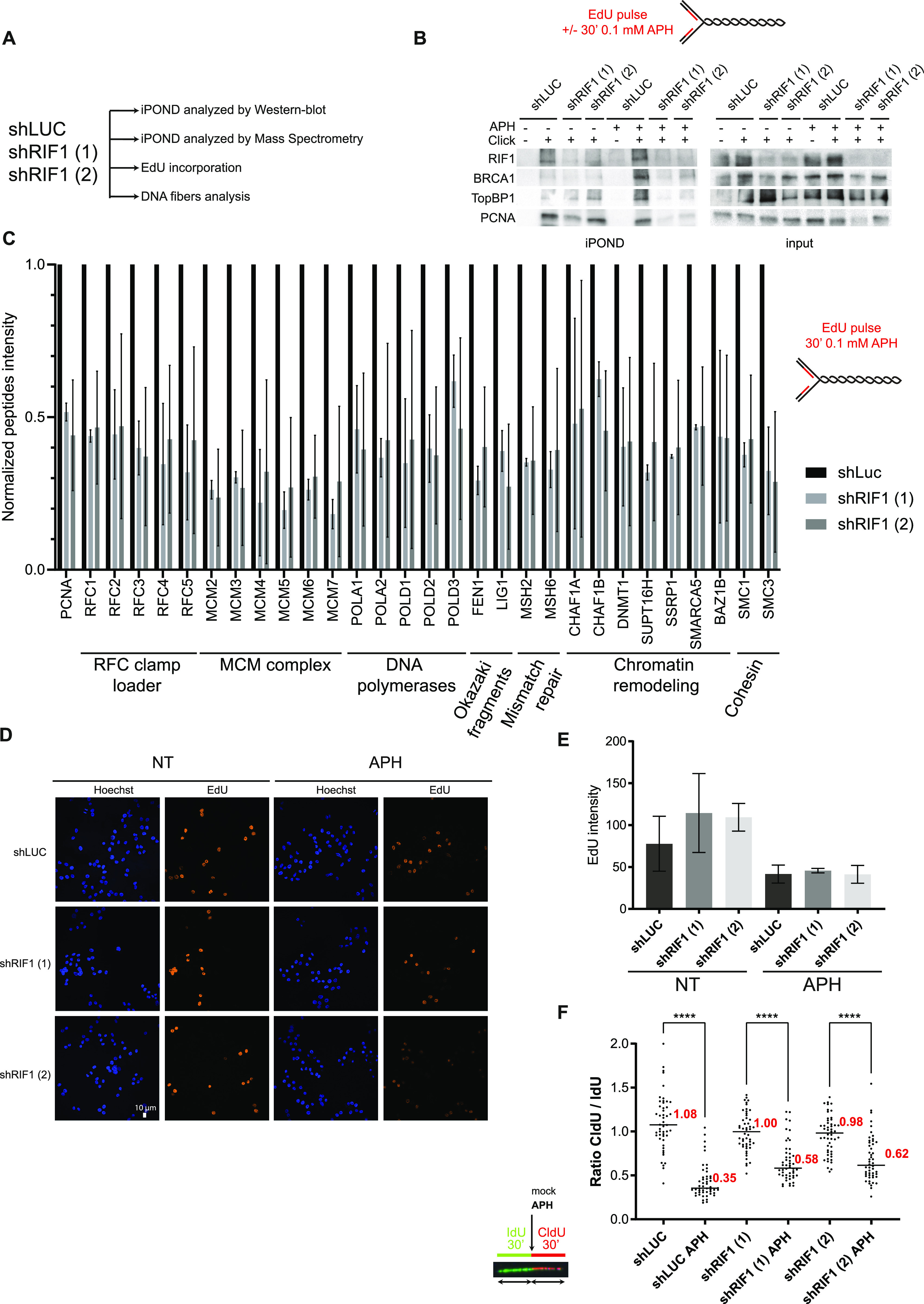
RIF1 loss reduces the efficacy of protein isolation on nascent DNA. **(A)** Experimental setup. **(B)** iPOND experiment. HeLa S3 cells (with shLUC or two different shRIF1) were labeled with EdU for 15 min or for 30 min with 0.1 μM aphidicolin (APH). Indicated proteins were analyzed by Western blotting. In no-click samples, biotin–TEG azide was replaced by DMSO. **(C)** iPOND-MS experiment. HeLa S3 cells (with shLUC or two different shRIF1) were labeled with EdU for 30 min EdU with 0.1 μM aphidicolin (APH). Quantification of peptide intensity for each protein was performed using MaxQuant; individual values are available in Table S2. The ratios of shRIF1 (1) or shRIF1 (2) versus shLUC are shown. The error bars represent the variation of two experiments for shRIF1 (1) and three experiments for shRIF1 (2). Because of normalization, there are no error bars for shLUC, but the experiment was performed three times. **(D)** Analysis of EdU incorporation using microscopy in HeLa S3 cells with shRNA against luciferase or RIF1. EdU was incorporated in cells during 15 min with or without 0.1 μM aphidicolin (APH). **(E)** Quantification of EdU intensity within nucleus stained with Hoechst was performed using CellProfiler and is represented on the histogram. Error bars correspond to the average values of three independent experiments. **(F)** DNA fiber labeling. HeLa S3 cells were labeled for 30 min with IdU and then for 30 min with CldU in the absence or presence of 0.05 μM aphidicolin (APH) in the cell culture medium. Graphic representation of the ratios of CldU versus IdU tract length. For statistical analysis, a Mann–Whitney test was used, ****P < 0.0001. The horizontal bar represents the median with the value indicated in red. At least 50 replication tracts were measured for each experimental condition.

**Figure S3.**
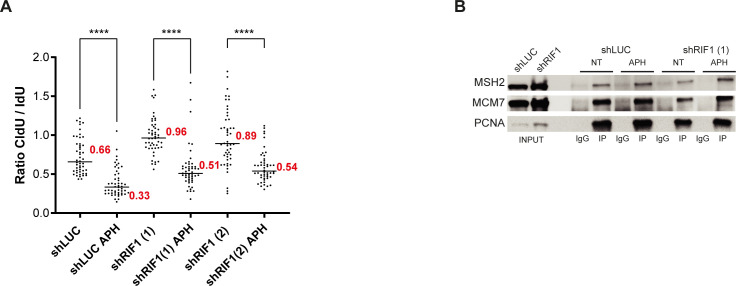
Treatment of RIF1 depleted-cells with aphidicolin does not perturb DNA synthesis or replisome stability. **(A)** Repetition of DNA fiber experiment from Fig 3F. HeLa S3 cells were labeled for 30 min with IdU and then for 30 min with CldU in the absence or presence of 0.05 μM aphidicolin (APH) in the cell culture medium. Graphic representation of the ratios of CldU versus IdU tract length. For statistical analysis, a Mann–Whitney test was used, ****P < 0.0001. The horizontal bar represents the median with the value indicated in red. At least 50 replication tracts were measured for each experimental condition. **(B)** Western blot analysis of indicated proteins after immunoprecipitation with an antibody directed against PCNA or against mouse IgG. When indicated, HeLa S3 cells (shLUC or shRIF1) were treated for 30 min with 0.1 μM aphidicolin (APH).

We showed that prolonged treatment with APH or thymidine yields high level of γH2A.X in RIF1-depleted cells. Importantly, after a thymidine block, the increase in γH2A.X signal correlates with alterations of DNA replication patterns. Because APH has also been widely used for cell synchronization, it is highly probable that the increased level of DNA lesions in the absence of RIF1 in APH-treated cells is also due to a defect in the maintenance of chromatin topology. Alternatively, the data could reflect a role of RIF1 in G1 cells rather than in the S phase. To understand this in more detail, we performed a series of experiments in RIF1-depleted cells using short treatments with aphidicolin (Fig 3A). First, we performed iPOND assay and probed isolated proteins by Western blotting (Fig 3B). Under standard cell culture conditions, the efficacy of PCNA isolation with nascent DNA in RIF1-depleted cells was similar to that of control cells (Fig 3B). As expected, a 30-min treatment with a low dose of APH (0.1 μM) induced the recruitment of BRCA1 and TopBP1 on nascent DNA (Fig 3B). Strikingly, in RIF1-depleted cells treated with APH, the efficacy of PCNA recovery with nascent DNA diminished dramatically (Fig 3B). To generalize this observation, we analyzed replisome composition using iPOND coupled to mass spectrometry in RIF1-depleted cells in response to APH (Fig 3C). In comparison with control cells, the treatment of RIF1-depleted cells with APH markedly reduced the abundance of well-established replication factors captured by iPOND (Lopez-Contreras et al, 2013) such as MCM subunits, RFC subunits, and proteins involved in Okazaki fragment maturation, mismatch repair, and chromatin remodeling (Fig 3C). However, this observation could be the consequence of a massive decrease in EdU incorporation that would impair protein recovery. To test this hypothesis, we measured EdU incorporation using immunofluorescence. As expected, APH treatment reduces strongly EdU incorporation, but this effect was similar in RIF1-depleted cells, suggesting that EdU incorporation is not impaired (Fig 3D and E). In addition, and consistent with Fig 1D, DNA fiber experiment performed in the exact same cell line than the one used for iPOND indicates that DNA synthesis is occurring, although at a lower pace, in response to APH independently of the presence of RIF1 (Figs 3F and S3A). Thus, a defect in DNA synthesis does not account for the reduced isolation of EdU-bound proteins from RIF1-depleted cells. Furthermore, we detected similar levels of the replisome-associated proteins MSH2 and MCM7 in PCNA immunoprecipitates from control and RIF1-depleted cells (Fig S3B), suggesting that RIF1 is not required for replisome stability and replication fork progression. At this step, the most reasonable hypothesis is that in the presence of replication stress, suppression of RIF1 reduces the efficacy of the capture of EdU-associated proteins.

### The efficacy of iPOND is biased by chromatin topology

**Figure 4.**
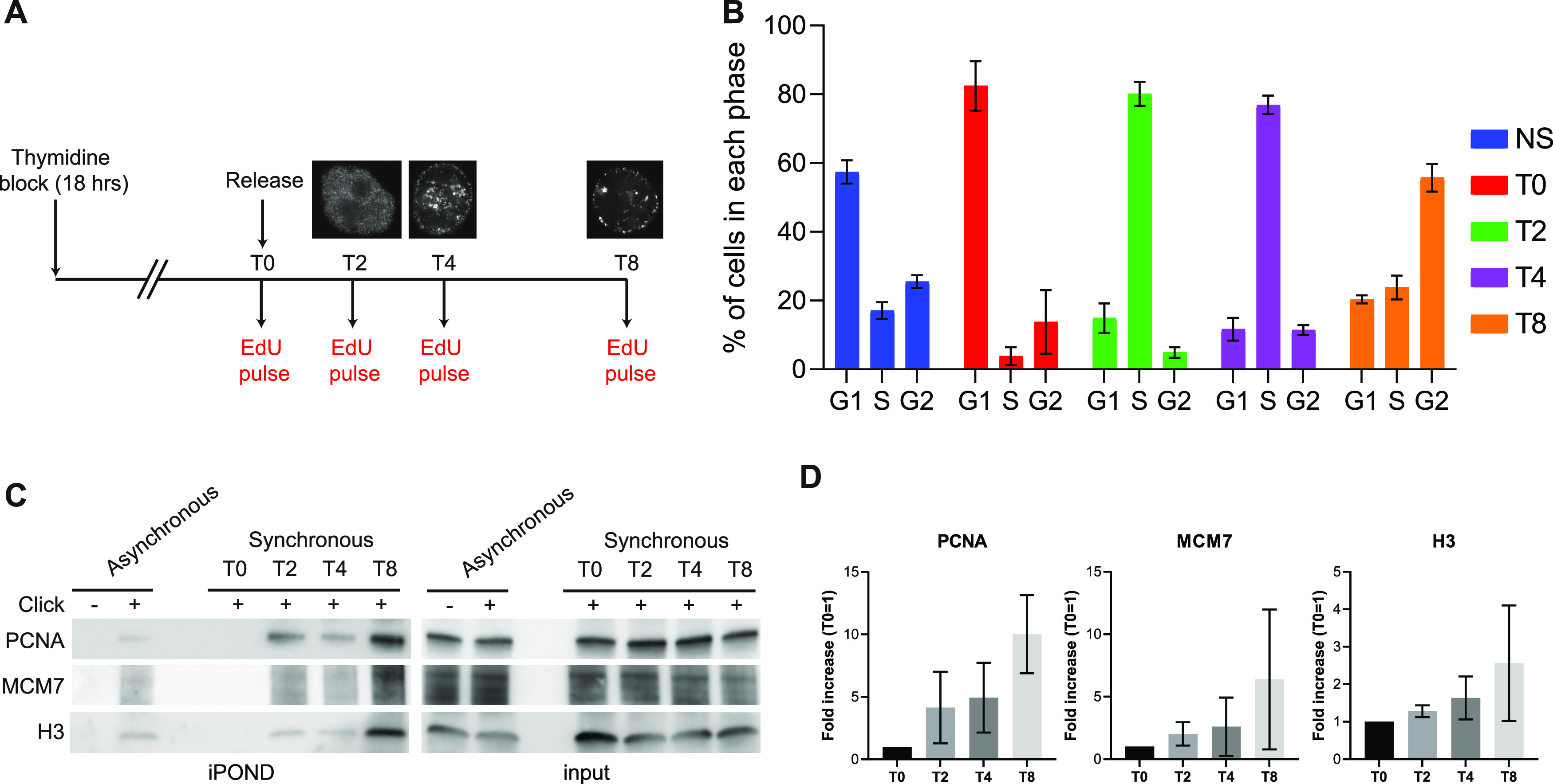
iPOND protein recovery is biased by replication organization. **(A)** Experimental setup. HeLa S3 cells were submitted to the thymidine block for 18 h and released into the S phase. Cells were collected at T0 (G1), T2 (Early-S), T4 (Mid-S), and T8 (Late-S) after a 15-min EdU pulse for iPOND and flow cytometry. Replication patterns showing the different phases are represented. **(B)** The percentage of cells in each phase was analyzed using flow cytometry. The error bars represent the variations within three independent experiments. **(C)** iPOND experiment performed on unsynchronized and synchronized cells and analyzed by Western blot using antibodies directed against the indicated proteins. In no-click samples, biotin–TEG azide was replaced by DMSO. **(D)** Quantification of the indicated proteins in iPOND based on at least three independent experiments; T0 was used for normalization.

**Figure S4.**
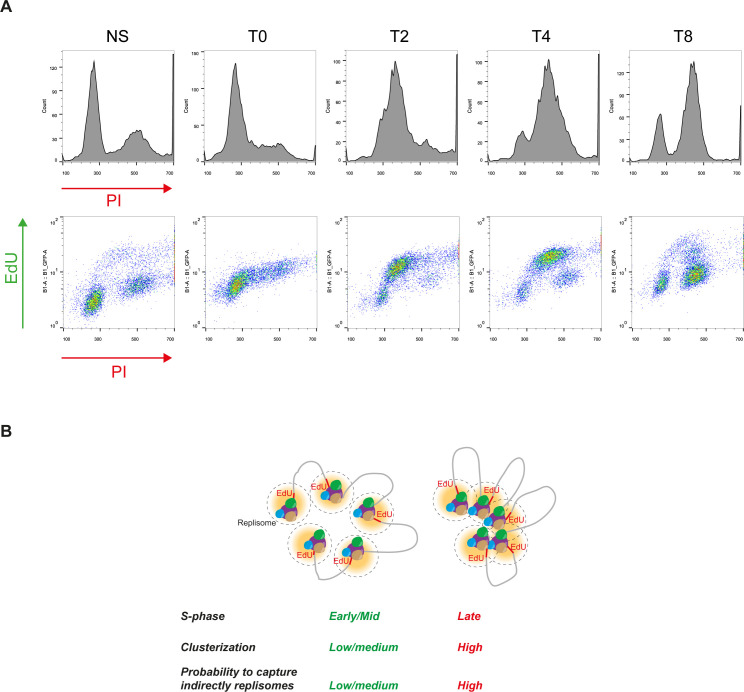
Putative impact of cell synchonization on replication factories. **(A)** Flow cytometry analysis of asynchronous HeLa S3 cells blocked for 18 h with thymidine (T0), then released for 2 (T2), 4 (T4), and 8 (T8) hours. Total DNA was stained using propidium iodide and newly synthesized DNA by EdU labeled with Alexa Fluor 488 using click chemistry. **(B)** Scheme explaining how replication organization is impacting iPOND efficiency.

How can we explain that in cells exposed to aphidicolin, RIF1 depletion decreases the efficacy of iPOND without affecting EdU incorporation? To answer to this question, one must take into consideration that the association of proteins such as cohesin or RIF1 with EdU may be indirect and determined by chromatin topology. Consistent with this, methods that are using formaldehyde crosslinking such as ChIP or chromosome conformation capture are indeed dependent on nuclear organization. To test whether iPOND efficiency is biased by chromatin organization, we took advantage of the distinct and characteristic patterns formed by replicons labeled with EdU (Dimitrova & Berezney, 2002). In the early S phase (replication of euchromatin), the EdU pattern is poorly clustered. Clusterization then increases in the mid-S phase (replication of facultative heterochromatin) and is even stronger in the late S phase (replication of constitutive heterochromatin). We synchronized HeLa S3 cells using a simple thymidine block procedure and released the cells in fresh media without thymidine (Fig 4A). We added EdU for 15 min just before release (T0) and then 2 h (T2), 4 h (T4), and 8 h (T8) after release (Fig 4A). We verified the synchronization procedure by flow cytometry using double labeling with EdU and propidium iodide (Figs 4B and S4A). As expected at T0, most (∼80%) of the cells were in G1. 2 and 4 h after release (T2 and T4), most of the cells (∼80%) were in the S phase. After 8 h (T8), cells entered G2 and the number of S-phase cells decreased (∼25%). We then performed iPOND experiment on synchronized and non-synchronized cells. At T0, the PCNA signal was barely detectable, as expected, and comparable to the control (minus click) of the asynchronous conditions (Fig 4C). In contrast, we could observe a clear PCNA signal after the EdU–biotin click reaction in the non-synchronized condition. At T2 and T4, the PCNA signal became detectable. Surprisingly, the strongest signal was observed at T8 even though the number of cells in the S phase is lower than in T4 and T2 (Fig 4C). This observation was also true for MCM7 and H3 (Fig 4C) and is reproducible (Fig 4D). This result indicates that the efficacy of protein isolation on nascent DNA does not correlate directly with the number of cells in the S phase. Therefore, we propose that the recovery of replisome components by iPOND may be influenced by the organization of replicated chromatin domains (Fig S4B).

### RIF1 depletion impairs the loading of SMC1 and SMC3 at forks in the presence of replication stress

**Figure 5.**
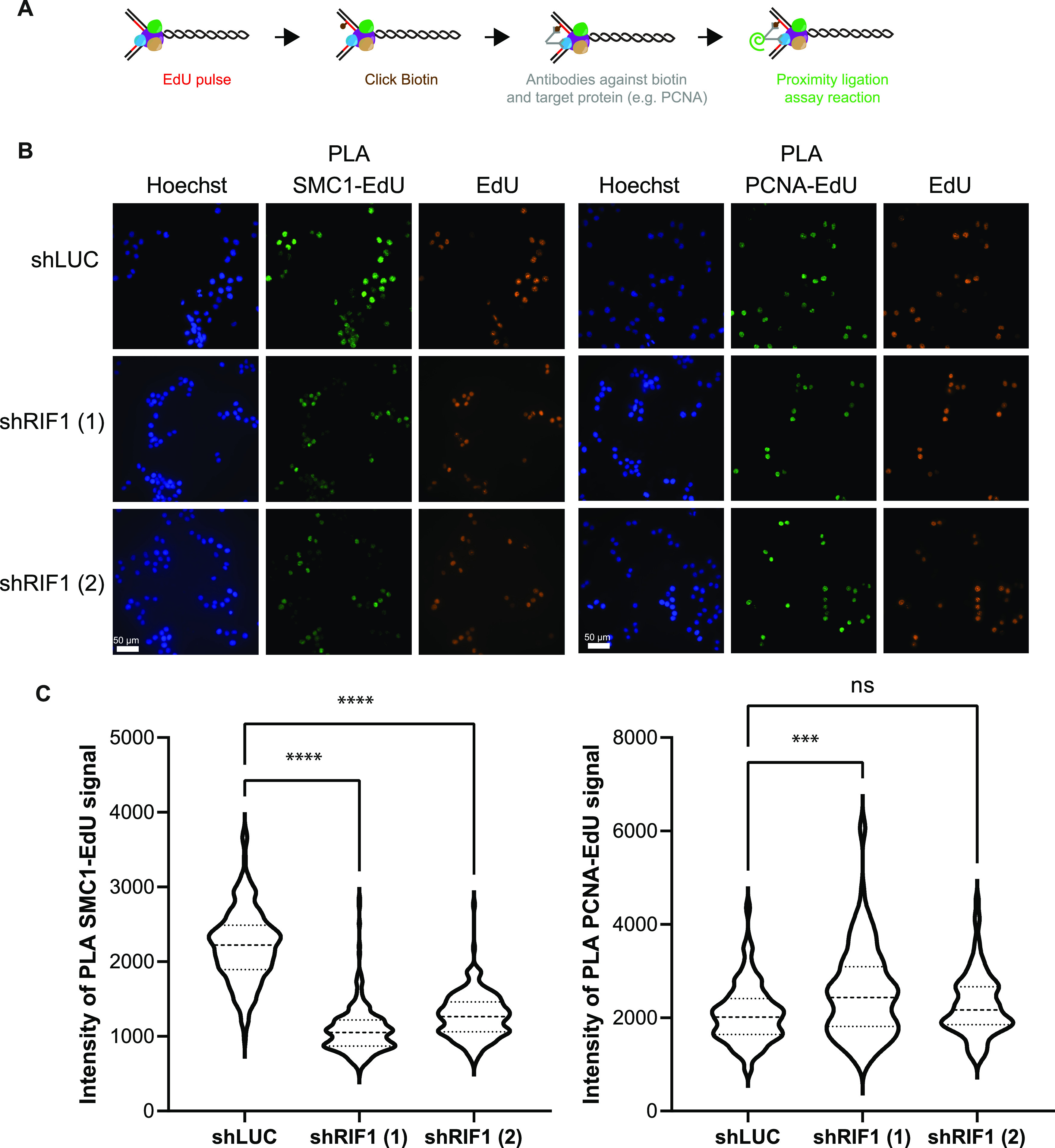
RIF1 is required for full recruitment of cohesin subunits at stalled forks. **(A)** Scheme explaining the principle of proximity ligation assay (PLA) between EdU and replisome components. **(B)** Immunofluorescence analysis of PLA signal between EdU and SMC1 and between EdU and PCNA upon 30-min treatment with 0.1 μM APH in HeLa S3 cells expressing shRNAs against luciferase or RIF1. EdU-positive cells were labeled with Alexa Fluor 555. **(C)** Level of PLA signal within the nucleus was quantified using CellProfiler. Graphic representation of the PLA signal; at least 100 cells were quantified in each condition. For statistical analysis, a Mann–Whitney test was used, ns, non-significant; ****P < 0.0001; and ***P < 0.001.

**Figure S5.**
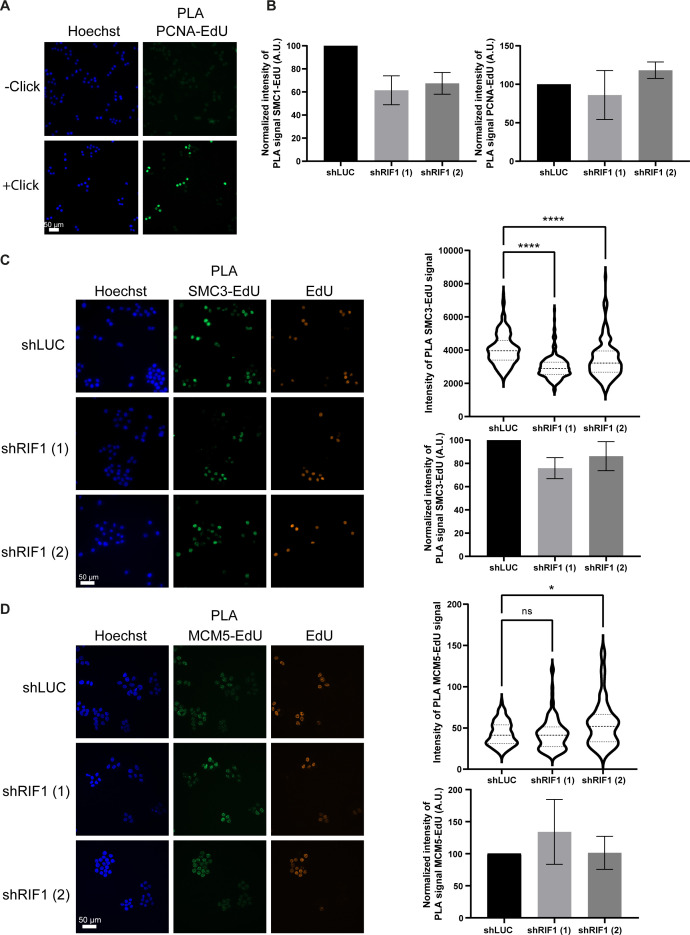
RIF1 is required for full recruitment of SMC3 at replication forks. **(A)** Immunofluorescence analysis of PLA signal between EdU and PCNA; in −click control, the biotin–TEG azide was replaced by DMSO. **(B)** Graphic representation of the average PLA signal (normalized to 100 in shLUC) from three independent experiments corresponding to Fig 5C. **(C)** Immunofluorescence analysis of PLA signal between EdU and SMC3 upon 30-min treatment with 0.1 μM APH in HeLa S3 cells expressing shRNAs against luciferase or RIF1. EdU-positive cells were labeled with Alexa Fluor 555. The level of PLA signal within the nucleus was quantified using CellProfiler. Graphic representation of the PLA signal; at least 100 cells were quantified in each condition. For statistical analysis, a Mann–Whitney test was used, ****P < 0.0001. Graphic representation of the average PLA signal (normalized to 100 in shLUC) from three independent experiments. **(D)** Immunofluorescence analysis of PLA signal between EdU and MCM5 upon 30-min treatment with 0.1 μM APH in HeLa S3 cells expressing shRNAs against luciferase or RIF1. EdU-positive cells were labeled with Alexa Fluor 555. The level of PLA signal within the nucleus was quantified using CellProfiler. Graphic representation of the PLA signal; at least 100 cells were quantified in each condition. For statistical analysis, a Mann–Whitney test was used, ****P < 0.0001. Graphic representation of the average PLA signal (normalized to 100 in shLUC) from three independent experiments.

**Figure S6.**
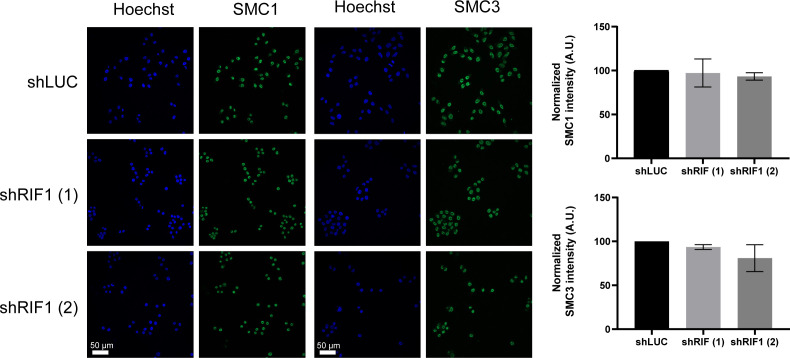
RIF1 depletion does not impact the expression of SMC1 and SMC3. Immunofluorescence analysis of SMC1 and SMC3. Graphic representation of the average SMC1 and SMC3 intensity within nucleus (normalized to 100 in shLUC) from three independent experiments.

RIF1 stabilizes chromatin topology via its intrinsic capacity to bridge molecules (Mattarocci et al, 2017) and may promote the recruitment of additional proteins involved in the organization of chromatin topology such as the cohesin complex. Indeed, cohesin subunits are associated with replication forks in basal conditions (Fig 1A) and in response to replication stress (Tittel-Elmer et al, 2012; Ribeyre et al, 2016). In addition, cohesin cooperates with RIF1 in the stabilization of chromatin topology at sites of DNA DSB (Ochs et al, 2019) and organizes DNA repair foci via a mechanism of loop extrusion at both sites of the DNA breaks (Arnould et al, 2021). Because RIF1 depletion diminishes the efficacy of the iPOND procedure (Fig 3C), we used, as an alternative method, a proximity ligation assay (PLA; Fig 5A) to analyze the loading of cohesin subunits in the vicinity of nascent DNA (Petruk et al., 2012, 2017; Roy et al, 2018). We first validated the method using PCNA as a positive control. As expected, we detected PCNA-EdU proximity signals in cells after the coupling of EdU and biotin, specifically (Fig S5A). We then analyzed the recruitment of SMC1 to nascent DNA in the presence of 0.1 μM APH. In control conditions, we observed a clear PLA signal between EdU and SMC1, confirming that SMC1 is recruited near stalled replication forks (Fig 5B). Interestingly, the signal of proximity between EdU and SMC1 was reduced in RIF1-depleted cells (Figs 5B and C and S5B). Consistent with this, the localization of SMC3 to stalled forks was also dependent on RIF1 (Fig S5C). In contrast, RIF1 suppression had no impact on EdU-PCNA proximity signal (Figs 5B and C and S5B) and EdU-MCM5 proximity signal (Fig S5D). We verified that the suppression of RIF1 did not reduce the level of SMC3 and SMC1 expression (Fig S6). Thus, we conclude that RIF1 contributes to the loading of the cohesin subunits SMC1 and SMC3 near stalled DNA replication forks in the presence of replication stress.

## Discussion

**Figure 6.**
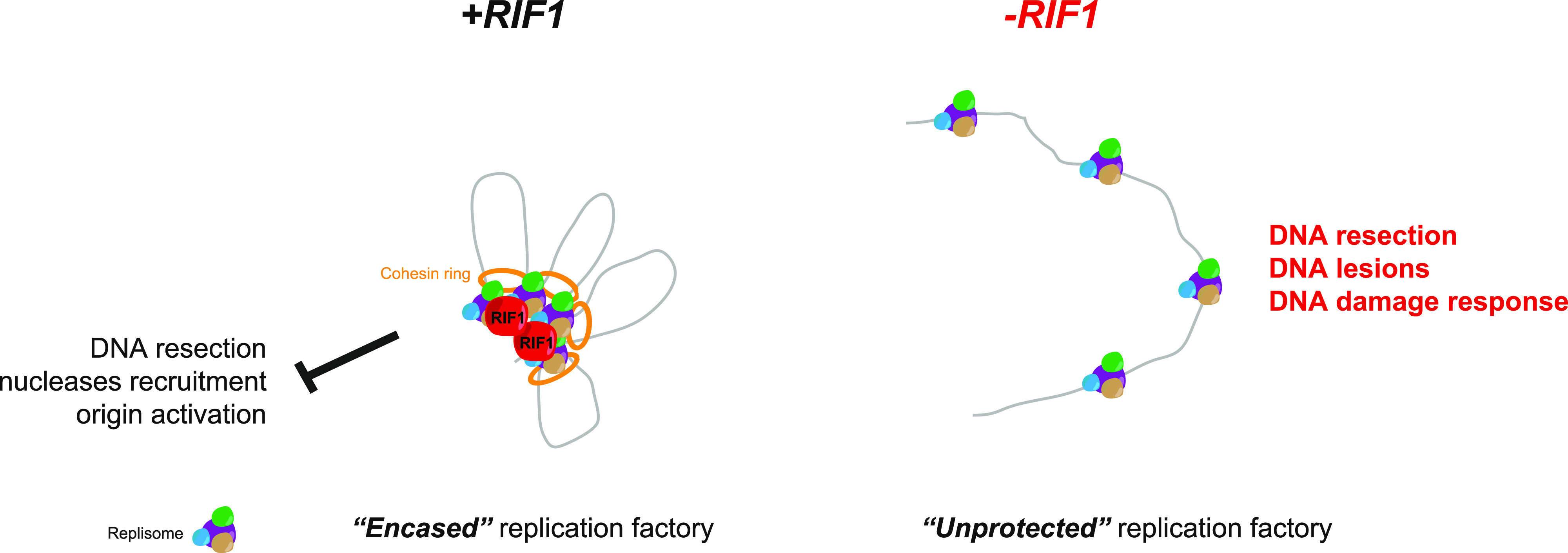
Putative model to explain the role of RIF1 in the organization of replication factories. RIF1 may stabilize chromatin topology during DNA replication, thus preventing DNA resection by nucleases or excessive origin activation. This could be direct, thanks to its capacity to interact with DNA, or/and via the recruitment of cohesin. In the absence of RIF1, the replication domains may be unprotected leading to DNA resection, DNA lesions, and activation of DNA damage response.

RIF1 was originally discovered more than 30 yr ago in budding yeast as a negative regulator of telomere elongation (Hardy et al, 1992). It is now clearly established that RIF1 is a highly conserved protein (Sreesankar et al, 2012) involved in telomere protection, DNA replication, DNA DSB repair, transcription, and heterochromatin formation (Mattarocci et al, 2016). The links between the seemingly disparate functions of RIF1 may stem from the function of RIF1 in the stabilization of chromatin topology (Arnould et al, 2021; Klein et al, 2021). Here, we provide evidence that the organization of chromatin architecture by RIF1 ensures chromosome stability during DNA replication stress. This model is based on the following findings: (1) RIF1 is localized near replication sites in basal conditions, (2) DNA replication stress in RIF1-depleted cells modifies S-phase patterns and increases the level of the DNA damage marker γH2A.X, (3) suppression of RIF1 strongly affects the organization of DNA replication in response to replicative stress, and (4) RIF1 may exert this function in coordination with the cohesin. Our model is consistent with the finding that RIF1 bridges proximal DNA molecules (Mattarocci et al, 2017) and creates a protective structure around DBSs (Ochs et al, 2019). Thus, we propose that the chromatin organizing function of RIF1 ensures DNA replication under stressful conditions (Fig 6). By analogy with its function at yeast telomeres, we would like to propose that RIF1 is protecting replication domains (Fig 6).

The association of RIF1 with the replication forks has been previously observed by other groups (Alabert et al, 2014; Her et al, 2018; Munden et al, 2018). We confirmed that suppression of RIF1 has no measurable effect on replication fork progression under standard conditions or in response to short treatment with replicative stress (Xu et al, 2010; Cornacchia et al, 2012; Ray Chaudhuri et al, 2016) despite the fact that RIF1 loss induces Chk1 phosphorylation on Ser345 (Chapman et al, 2013; Foti et al, 2016). Interestingly, we found that 2 h after release from a thymidine block, replication tracts are longer in the absence of RIF1 and phosphorylation levels of Chk1 on Ser345 and H2A.X on Ser139 are increased. One possibility is that in the absence of RIF1, the disorganization of chromatin domains during DNA replication results in the accumulation of single-stranded DNA. The accumulation of DNA lesions likely underpins the increased sensitivity of RIF1-defective cells to inhibitors of DNA replication (Buonomo et al, 2009; Xu et al., 2010, 2017; Feng et al, 2013).

RIF1 is recruited by 53BP1 at DSBs to prevent homologous recombination and favor NHEJ (Chapman et al, 2013; Di Virgilio et al, 2013; Escribano-Diaz et al, 2013; Zimmermann et al, 2013). Based on this, it has been proposed that RIF1 could be recruited by 53BP1 to protect stalled forks independently of BRCA1 (Xu et al, 2017). These data are raising the possibility that 53BP1 contributes to the recruitment of RIF1 at replication forks in basic conditions and in response to replicative stress. However, RIF1 recruitment is not impacted by 53BP1 depletion (Her et al, 2018) and RIF1, but not 53BP1, protects nascent DNA from degradation (Ray Chaudhuri et al, 2016), suggesting that the presence of RIF1 at replication forks is independent of 53BP1, consistent with its capacity to form higher order structures in budding yeast (Mattarocci et al, 2017).

Our model is consistent with the observation that RIF1 protects stalled replication forks from resection by nucleases, perhaps via the creation of a compartment that prevents their recruitment (Ray Chaudhuri et al, 2016; Garzón et al, 2019; Mukherjee et al, 2019) possibly thanks to the phosphorylation of its intrinsically disordered region (Balasubramanian et al, 2022). A role of RIF1 in safeguarding the stability of replicated domains may also explain how RIF1 controls the activation of dormant origins in response to replicative stress (Hiraga et al, 2017) and prevents the formation of anaphase bridges (Hengeveld et al, 2015; Zaaijer et al, 2016). RIF1 depletion has a strong impact on replication timing (Cornacchia et al, 2012; Yamazaki et al, 2012; Foti et al, 2016; Richards et al, 2022). The action of RIF1 on the replication timing program may result from the regulation of DDK kinase activation through RIF1 interaction with the PP1 phosphatase (Dave et al, 2014; Hiraga et al, 2014; Mattarocci et al, 2014) or through its ability to bind G-quadruplexes and to organize chromatin topology (Kanoh et al, 2015). Because the loss of RIF1 induces drastic changes in nuclear organization revealed by chromosome conformation capture methods (Foti et al, 2016), we favor the hypothesis that the impact of RIF1 on replication timing is a consequence of impaired nuclear organization rather than of a defect in the control of DDK kinases. The latter hypothesis is supported by recent evidence based on Hi-C chromosome conformation capture experiments, showing that RIF1 is necessary to enforce chromosome interaction hubs that determine the replication timing program (Klein et al, 2021). This model could explain why suppression of RIF1 perturbs transcription and heterochromatin formation (Dan et al, 2014; Hiraga et al, 2017; Klein et al, 2021). Because the recruitment of cohesin at stalled forks is dependent on RIF1, it is tempting to speculate that RIF1 might ensure the stabilization of replicating chromatin domains in coordination with cohesin. Consistent with this, the depletion of cohesin subunits mimics topological alterations at DSBs caused by the depletion of RIF1 (Ochs et al, 2019). In contrast, induced degradation of SCC1 did not impact the patterns of replication (Oldach & Nieduszynski, 2019). We favor a model where the cohesin complex is recruited by RIF1 directly at stalled forks to maintain the local organization of chromatin, with no impact on the general organization of DNA replication (Tittel-Elmer et al, 2012; Ribeyre et al, 2016).

Finally, this study illustrates a yet unforeseen application of iPOND (or iPOND-related methods based on formaldehyde crosslinking). It is generally assumed that the iPOND method captures proteins associated with individual replisomes distributed along a linear DNA template. Here, we show that the iPOND method is efficient not only to isolate replisome components but also to capture structural components of replicating chromatin domains stabilized by formaldehyde crosslinking. Future studies using iPOND and other methods should provide new insights into the role of the nuclear organization in DNA replication.

## Materials and Methods

### Cell lines

HeLa S3 (obtained from ATCC) cells were cultured in DMEM. Culture media were supplemented with 10% fetal bovine serum (Biowest) and penicillin/streptomycin (Sigma-Aldrich). Cells were incubated in 5% CO_2_ at 37°C. For thymidine block experiments, cells were treated 18 h with 2 mM thymidine, washed, and then released into normal media.

### Gene silencing

For RIF1 depletion, siRNA oligonucleotides were purchased from Dharmacon (M-027983-01-0005) and transfected using INTERFERin (Polyplus-transfection). Anti-RIF1 shRNAs (1) and (2) anti-luciferase shRNA were cloned in pSUPER-EBV and transfected using Lipofectamine 2000 (Thermo Fisher Scientific). Stable cell lines were selected using puromycin.

### Western blot

The proteins were resolved by SDS–PAGE using homemade or precast gels (Bio-Rad) and transferred to a nitrocellulose membrane (GE Healthcare or Bio-Rad). Antibodies against the following proteins were used: Ser345 phospho-Chk1 (2348; Cell Signaling Technology), Chk1 (sc-8408; Santa Cruz), PCNA (P8825; Sigma-Aldrich), Ser4/8 phospho-RPA32 (A300-245A), RPA32 (NA18; Calbiochem), TopBP1 (A300-111A; Bethyl), histone H3 (ab62642; Abcam), BRCA1 (sc-642; Santa Cruz), RIF1 (A300-568A-M; Bethyl), and MCM7 (ab2360; Abcam).

### Co-immunoprecipitation

Cells were incubated for 30 min in ice in high salt buffer (50 mM Tris, pH 7.5, 300 mM NaCl, 1% Triton, and 1 mM DTT). After 10-min centrifugation at 14,000*g*, the supernatant was incubated with anti-PCNA antibody (P8825; Sigma-Aldrich) or IgG rabbit (NI01; Calbiochem) overnight at 4°C. Magnetic beads coupled with protein G (10004D; Life) were added for 1 h and washed five times with washing buffer (10 mM Hepes, 100 mM KOAc, and 0.1 mM MgOAc). Beads were boiled in Laemmli buffer, and supernatants were analyzed by Western blot.

### iPOND

iPOND was performed largely as described in Lossaint et al (2013) and Ribeyre et al (2016). Briefly, HeLa S3 cells were pulse-labeled with 10 μM EdU for 5–15 min and a 120-min chase was performed with 10 μM thymidine. Cells were fixed with 1% formaldehyde for 5 min followed or not by quenching of formaldehyde by 5-min incubation with 0.125 M glycine. Fixed samples were collected by centrifugation at 1,000*g* for 3 min, washed three times with PBS, and stored at −80°C. Cells were permeabilized with 0.5% Triton, and click chemistry was used to conjugate biotin–TEG azide (Eurogentec) to EdU-labeled DNA. Cells were resuspended in lysis buffer, and sonication was performed using a Qsonica sonicator. Biotin-conjugated DNA–protein complexes were captured using streptavidin beads (Ademtech). Captured complexes were washed with lysis buffer and high salt. Proteins associated with nascent DNA were eluted under reducing conditions by boiling into SDS sample buffer for 30 min at 95°C.

### DNA fiber labeling

DNA fiber labeling was performed as previously described (Lossaint et al, 2013; Ribeyre et al, 2016). Cells were labeled with 25 μM IdU, washed with warm media, and exposed to 50 μM CldU. Cells were lysed, and DNA fibers were stretched onto glass slides. The DNA fibers were denatured with 2.5 M HCl for 1 h, washed with PBS, and blocked with 2% BSA in PBS/Tween for 60 min. IdU replication tracts were revealed with a mouse anti-BrdU/IdU antibody from BD Biosciences (347580) and CldU tracts with a rat anti-BrdU/CldU antibody from Eurobio (ABC117-7513). The following secondary antibodies were used: Alexa Fluor 488 anti-mouse antibody (A11001; Life) and Cy3 anti-rat antibody (712-166-153; Jackson ImmunoResearch). Replication tract lengths were analyzed using ImageJ software. For statistical analysis, we used a non-parametric Mann–Whitney test with Prism software.

### Immunofluorescence

Cells were plated on glass coverslips and fixed with 4% paraformaldehyde in PBS for 20 min at room temperature. When indicated, cells were incubated with EdU (5-ethynyl-2′-deoxyuridine) for the indicated times. PFA-fixed cells were permeabilized with 0.2% Triton X-100 in PBS for 5 min. Primary (Ser139 phospho-H2A.X; Millipore, 05-636 and RIF1; Bethyl, A300-568A-M) and secondary (anti-mouse Alexa Fluor 488 and anti-rabbit Alexa Fluor 546) antibodies were prepared in PBS with 0.1% Tween, and incubations were carried out in a humidified chamber at room temperature (60 and 30 min, respectively). EdU was coupled with Alexa Fluor 555 using click chemistry. DNA was stained with Hoechst. The cells were mounted on glass slides with Prolong (Life). Cells were analyzed by fluorescence microscopy, and quantification of various signals was performed using CellProfiler software (Carpenter et al, 2006).

### Proximity ligation assay

Cells were plated on glass coverslips and fixed with 4% paraformaldehyde in PBS for 20 min at room temperature. When indicated, cells were incubated with EdU (5-ethynyl-2′-deoxyuridine). PFA-fixed cells were permeabilized with 0.5% Triton X-100 in PBS for 20 min. EdU was coupled with Alexa Fluor 555 or biotin–TEG azide using click chemistry. Primary antibodies against SMC1 (A300-055A; Bethyl), SMC3 (A300-060A; Bethyl), MCM5 (17967; Abcam), PCNA (P8825; Sigma-Aldrich), and biotin (A150-109A; Bethyl or 200-002-211; Jackson ImmunoResearch) were incubated overnight. Probes from Duolink In Situ PLA Probe Anti-Rabbit PLUS (DUO92002; Sigma-Aldrich) and Duolink In Situ PLA Probe Anti-Mouse MINUS (DUO92004; Sigma-Aldrich) were incubated with coverslip for 60 min at 37°C. For ligation (30 min at 37°C) and amplification (100 min at 37°C), Duolink In Situ Detection Reagents Green (DUO92014; Sigma-Aldrich) was used. The cells were mounted on glass slides with Duolink In Situ Mounting Medium with DAPI (DUO82040; Sigma-Aldrich). Cells were analyzed by fluorescence microscopy, and quantification of PLA signal was performed using CellProfiler software (Carpenter et al, 2006).

### Flow cytometry

Cells were labeled with EdU for 15 min, then fixed in 80% ethanol. After permeabilization, EdU was coupled with Alexa Fluor 488 using click chemistry. DNA was stained using propidium iodide, and analysis was performed on a Miltenyi MACSQuant device.

### Mass spectrometry analysis

Mass spectrometry was performed as indicated in Kumbhar et al (2018) or using the following protocol. Protein digestion was performed using S-Trap micro columns (ProtiFi) following the manufacturer’s instructions. Briefly, after reduction (DTT 20 mM, 10 min at 95°C) and alkylation (IAA 40 mM, 30 min at RT) proteins were digested using trypsin (1 μg/sample, 2 h at 47°C; Gold, Promega). For LC–MS/MS analysis, peptides were loaded onto a 25-cm reversed-phase column (75 mm inner diameter; Acclaim PepMap 100 C18; Thermo Fisher Scientific) and separated with an UltiMate 3000 RSLC system (Thermo Fisher Scientific) coupled to a Q Exactive HFX system (Thermo Fisher Scientific). Separation of the peptides was performed following a gradient from 2% to 25% buffer B (0.1% AF in 80% ACN) for 40 min at a flow rate 300 nl/min, then 25–40% for 20 min, and finally 40–90% for 2 min. Tandem mass spectrometry analyses were performed in a data-dependent mode. Full scans (350–1,500 m/z) were acquired in the Orbitrap mass analyzer with a resolution of 120,000 at 200 m/z. For MS scans, 3e6 ions were accumulated within a maximum injection time of 60 ms. The 20 most intense ions with charge states ≥2 were sequentially isolated (1e5) with a maximum injection time of 50 ms and fragmented by higher energy collisional dissociation (normalized collision energy of 28) and detected in the Orbitrap analyzer at a resolution of 30,000. Analysis of raw files was performed using MaxQuant (Cox & Mann, 2008), version 1.5.6.5, using default settings with label-free quantification option enabled. Raw file spectra were searched against the human UniProt reference database. Protein, peptide, and site false discovery rate were adjusted to < 0.01.

## Supplementary Material

Reviewer comments
